# Prevention of Mental Health Difficulties for Children Aged 0–3 Years: A Review

**DOI:** 10.3389/fpsyg.2020.500361

**Published:** 2021-09-29

**Authors:** Elizabeth Izett, Rosanna Rooney, Susan L. Prescott, Mia De Palma, Maryanne McDevitt

**Affiliations:** ^1^School of Psychology, Faculty of Health Sciences, Curtin University, Perth, WA, Australia; ^2^The ORIGINS Project, Telethon Kids Institute and the Division of Paediatrics, School of Medicine, University of Western Australia, Nedlands, WA, Australia

**Keywords:** early intervention, parent mental health, early childhood mental health, infants and toddlers, intervention and prevention, parenting programs, zero to three

## Abstract

The period of infancy and early childhood is a critical time for interventions to prevent future mental health problems. The first signs of mental health difficulties can be manifest in infancy, emphasizing the importance of understanding and identifying both protective and risk factors in pregnancy and the early postnatal period. Parents are at a higher risk of developing mental health problems during the perinatal period. An understanding of the evidence around prevention and intervention for parental anxiety and depression is vital to the process of prevention of early mental health disorders in infants and young children. Here we review the existing prevention and treatment interventions in the early years focusing on the period from conception to 3 years – the majority targeting parents in order to improve their mental health, and that of their infants. Elements of successful programs for parents include psychoeducation and practical skills training, as well as work on the co-parenting relationship, developing secure attachment, and enhancing parental reflective functioning. While both targeted and universal programs have produced strong effect sizes, universal programs have the added benefit of reaching people who may otherwise not have sought treatment. In synthesizing this information, our goal is to inform the development of integrated models for prevention and novel early intervention programs as early in life as possible.

## Introduction

Across the world, 10–20% of children and adolescents suffer from mental health disorders (Kessler et al., 2007). If not treated, mental health disorders of childhood have a severe, negative impact on children’s development and their long-term capacity to live healthy, productive and fulfilling lives (Lyons-Ruth et al., 2017). Mental health disorders have a profound impact on all aspects of health, happiness and productivity. The personal, social and economic costs of mental health difficulties are high, including clinical and education services, sick-leave and unemployment, and the criminal justice system (Bor et al., 2004). The direct and indirect economic cost of mental illness in Australia has been estimated at $20 billion per year, which includes the cost of lost productivity and labor force participation (Department of Health and Ageing, 2007). The burden of illness from mental health disorders accounts for approximately 12% of the total disability-adjusted life years associated with all illnesses, the third highest after cancer and cardiovascular diseases (Australian Institute of Health and Welfare, 2016).

As mental health difficulties early in life have a significant impact on the future health trajectory of an individual, it is essential to build the foundations of good mental health during this critical period (Lewis et al., 2014; Moore et al., 2017). In this review, we consider interventions offered at the earliest opportunity spanning from the perinatal period to 3 years of age aimed at preventing mental health difficulties in childhood. We begin with a description of mental health in infancy and early childhood and go on to present the current prevalence statistics within the population. We then consider the factors that contribute to, or detract from, good mental health during infancy and early childhood, including parent-child attachment, parental reflective functioning, parental mental health, co-parenting and the couple relationship as well as childhood experiences – recognizing that these factors can be used to inform prevention and early intervention efforts (Bayer et al., 2008). Interventions that aim to improve parent mental health during this period are also considered, given that parental mental health has been found to be a risk factor for poor outcomes in infants and children, including emotional and conduct problems later in life (Bauer et al., 2016). Finally, we explore the current approaches to prevent mental health problems during infancy, and how these might be extended.

## Mental Health Problems in Infancy and Early Childhood

The current statistics indicate an alarming rate of mental health problems in children and adolescents. As noted above, 10–20% of children and adolescents experience mental health problems worldwide (World Health Organization, 2003). While it is widely accepted that we are in the midst of a mental health crisis for young people, what is often missed is that the precursors of mental health problems can begin as early as the perinatal period and early infancy (Robinson et al., 2008). This makes the perinatal, infant and early childhood period a crucial window for intervention, with the goal of promoting good mental health for infants and young children (Robinson et al., 2008).

Good mental health in infancy and early childhood refers to healthy social and emotional development. It includes an infant’s ability to experience, regulate and express emotions, to develop close and secure interpersonal relationships, and to explore the environment and learn (Clinton et al., 2016). All of these capacities develop best within the context of a caregiving environment that includes family, community, and cultural expectations for young children (Parlakian and Seibel, 2002).

Research has established that infants and toddlers can suffer from mental health disorders that require treatment in their own right (Warner and Pottick, 2006; Zero to Three, 2012). Difficulties in infancy include regulatory disturbances such as excessive crying, sleeping or feeding difficulties and attachment difficulties (Postert et al., 2012; ZERO TO THREE, 2016). Early childhood mental health problems include externalizing problems such as aggression and oppositional defiance (Egger and Angold, 2006; Loeber et al., 2009), and internalizing problems such as anxiety and depression (Costello et al., 2005; Rapee et al., 2009; Bayer et al., 2011).

Several epidemiological studies have determined the prevalence of mental health disorders in infants and young children, indicating a 16–18% prevalence of mental health disorders amongst children aged 1–5 years, with approximately half of these children being severely impacted (von Klitzing et al., 2015). One birth cohort study found that almost 35% of infants aged 12–18 months scored high on the Problem Scale of the Brief Infant Toddler Social Emotional Assessment (BITSEA) (Horwitz et al., 2013), while another study found that by 18 months of age, 16–18% of children met criteria for one or more diagnoses of a mental health or developmental disorder (Skovgaard et al., 2007). Similar results were reported by an Australian study, which found that by 2 years of age, 12% of children had clinically significant emotional, behavioral, or social problems in the context of parent-child relationship disturbance (Bayer et al., 2011). In addition to this, a Western Australian study found that by age five, 20% of the children studied had clinically significant behavioral problems (Robinson et al., 2008). As noted by Lyons-Ruth et al. (2017), most recent epidemiological studies have been conducted in developed, economically stable, peaceful countries. However, evidence suggests that rates of mental health difficulties may be much higher in countries where extreme poverty, war, family displacement and trauma exist (Tomlinson et al., 2014).

Mental health problems in infants and toddlers are strongly predictive of poor mental, physical, cognitive, and social outcomes during childhood, adolescence and adulthood. Regulatory problems in infancy are associated with later motor, language and cognitive delays, behavioral problems and ongoing parent-child relationship difficulties (DeGangi et al., 2000; Hemmi et al., 2011; Cook et al., 2019). In one longitudinal study, when children were followed up to the their early schooling, two-thirds of the infants and toddlers (aged 12 to 40 months) who had earlier emotional and behavioral difficulties continued to have ongoing difficulties with persistent psychopathology (Briggs-Gowan et al., 2006). Another study found difficult temperament, non-compliance and aggression in infancy and toddlerhood (age one to three years) was associated with internalizing and externalizing psychiatric disorders at five years of age (Keenan et al., 1998).

If not treated, mental health difficulties that begin early in life can become more serious over time (Lavigne et al., 1998; Shaw et al., 2003; Briggs-Gowan et al., 2006; Suveg et al., 2007; Slemming et al., 2010; Clinton et al., 2016), and can persist into adolescence and adulthood (Bor et al., 2004; National Scientific Council on the Developing Child, 2008; Bayer et al., 2011). Children with mental health problems are at higher risk for difficulties at school, difficulties with peers, difficulty participating in employment, drug and alcohol problems, relationship breakdown, family violence, criminal activity, juvenile delinquency and suicide (Bayer et al., 2008; National Scientific Council on the Developing Child, 2008). Consequently it is vital to provide interventions that aim to prevent mental health difficulties from developing at this early age.

## Risk Factors for Infant and Early Childhood Mental Health Problems

There is international consensus that the first 1,000 days of life – the period of development from conception to age two – represent a crucial period of rapid physical, psychological and neurological growth (Moore et al., 2017). During this time there is an increased likelihood that detrimental experiences such as early trauma or deprivation will be especially harmful and greatly impact future development, with adverse effects potentially going on to develop into lifelong consequences (Shonkoff et al., 2012; Lyons-Ruth et al., 2017; Moore et al., 2017).

Researchers have identified several risk factors for poor mental health in infants, taking into account the interaction between the individual child’s genetics, temperament and environment (McLuckie et al., 2019). Risks in the child include the presence of physical health difficulties, a difficult temperament, and insecure and disorganised attachment patterns (Bosquet and Egeland, 2006; Van Zeijl et al., 2006; Miner and Clarke-Stewart, 2008; Edwards et al., 2010; Wlodarczyk et al., 2017). Family based risk factors include parenting interactions that are insensitive, lack warmth, or are controlling, as well as parental interactions that are over involved or over protective. Other factors include overly harsh discipline, parental mental health difficulties or stress, parental substance abuse, family violence, limited parental education, and parental conflict or separation/divorce (Dwyer et al., 2003; McCarty et al., 2005; Bayer et al., 2006, 2011; Pike et al., 2006; Van Zeijl et al., 2006; Ashford et al., 2008; Miner and Clarke-Stewart, 2008; Edwards et al., 2010; Wlodarczyk et al., 2017).

The experience of trauma during infancy, including maltreatment, exposure to domestic violence, and disruption of attachment relationships are significant developmental risk factors that lead to vulnerabilities to later mental health difficulties (Newman et al., 2016). The Adverse Childhood Experiences (ACE) study found that trauma that occurs early in development is associated with an increased risk of mental health problems and chronic diseases later in life including depression, suicide attempts, substance use, teen pregancy, heart disease, cancer, lung disease and liver disease (Felitti et al., 1998; Campbell et al., 2014). Furthermore, this level of risk increases exponentially with increased adverse childhood experiences. Researchers have found that individuals with four or more adverse childhood experiences are at significantly higher risk of developing a mental health problem than individuals with none (Felitti et al., 1998). Epigenetic studies have found that early childhood maltreatment is linked to higher methylation of key genes related to the stress response. These methylation changes place children at risk of cancers, cardiovascular disease, autoimmune disorders, and psychiatric disorders (Perry, 2004; Cicchetti et al., 2016). Environmental stressors such as war, natural disasters and family dislocation also place infants and young children at risk of mental health difficulties, especially if the primary caregiver is rendered less emotionally available by the same stressor (Lyons-Ruth et al., 2017).

## Parent Depression and Anxiety During the Perinatal and Infancy Periods

The perinatal period is a vulnerable time for parent stress and mental health difficulties (Jones et al., 2014). Perinatal depression affects approximately 11.9% of pregnant women worldwide (Woody et al., 2017). This value is from a study of pooled prevalence analyzing 96 prevalence studies from across the globe (Woody et al., 2017). Postnatal depression is associated with a disruption in the parent-infant attachment relationship which can place the infant at increased risk of mental health difficulties (Lyons-Ruth et al., 2017), it is also related to poor outcomes in cognitive and emotional development of children, and increased risk of psychopathology (Kingston et al., 2012). Anxiety related disorders are also prevalent during the perinatal period, with approximately 15% of women experiencing anxiety (Fairbrother et al., 2016). Perinatal anxiety is also related to poor outcomes in children, with increased incidence of internalizing disorders, somatic problems and behavioral inhibition found in children whose mothers experienced anxiety related disorders in the perinatal period (Bauer et al., 2016). Another meta-analysis focused on comorbid depression and anxiety during the perinatal period (Falah-Hassani et al., 2017). This is noteworthy as comorbid disorders tend to present a higher risk than single disorders. The prevalence of comorbid depression and anxiety symptoms during the perinatal period was 8.1%, and the prevalence of a diagnosed comorbid disorder was 7.9% (Falah-Hassani et al., 2017). These results indicate that comorbid depression and anxiety impact one in ten women during pregnancy and one in twelve women during the postnatal period (Falah-Hassani et al., 2017).

Perinatal depression in fathers has been less researched but is being increasingly recognized as important for ongoing child wellbeing (Philpott and Corcoran, 2018). A meta-analysis found that rates varied across studies, but the average prevalence of depression in fathers across the perinatal period was 8.4%, with similar rates in the prenatal and postnatal periods (Cameron et al., 2016). This is higher than the rate of depression in the general population (Philpott and Corcoran, 2018). It is important to note that the prevalence of perinatal depression in fathers was not much lower than the average prevalence for mothers (Woody et al., 2017). There is also a positive correlation between maternal and paternal depression (Cameron et al., 2016; Philpott and Corcoran, 2018).

There are a number of risk and protective factors that have been identified for perinatal depression and anxiety in parents. Risk factors include a history of depression and anxiety, lack of support and difficulty bonding with the new baby (PANDA, 2017). Mothers who have difficulty soothing their baby and getting them to sleep have a higher risk of mental health problems. Parents whose partners are depressed are also at higher risk of developing perinatal depression (Cameron et al., 2016). Parental anxiety and depression pose significant risks to the developing infant’s mental health (Bauer et al., 2016). Poor parental mental health during the transition to parenthood can have long lasting consequences for the infant, impacting their developmental trajectory and future mental health and wellbeing (Bauer et al., 2016). Consequently, programs aimed at enhancing mental health in infancy and early childhood should focus on parent mental health, as parent wellbeing during this time plays an important role in ensuring the positive mental health of their children.

Cognitive-behavioral therapy and interpersonal psychotherapy have been identified in a review as effective treatment approaches for pregnant women with major depressive disorder, suggesting that interventions should be based on these approaches (Van Ravesteyn et al., 2017).

A review of prevention programs for postnatal depression found that those using interpersonal therapy were the most consistent in delivering effective results (Werner et al., 2016). Mixed results were seen for cognitive behavioral programs, although individual interventions appeared more effective than group-based interventions (Werner et al., 2016).

Cognitive Behavioral interventions in the perinatal period have been found to be an effective intervention treatment for depression (Gloaguen et al., 1998; Barkham et al., 1999; Sockol, 2015; Ziemeng et al., 2020; Holt et al., 2021). These interventions have typically targeted evaluating potentially problematic styles such as catastrophic thinking and including behavioral strategies such as relaxation to assist in coping with stressful situations.

## Attachment and Mentalization

Attachment theory asserts that the relationship between the infant and their primary carers has an important influence on the development of the capacity for emotional and behavioral regulation (Bowlby, 1969; Ainsworth et al., 1978). A large body of evidence has identified that an infant’s developing brain is shaped by the quality of the caregiving environment provided by their primary caregivers (Kerns and Brumariu, 2014; Lally and Mangione, 2017). Secure primary attachment relationships, although not a guarantee against future mental health difficulties, are influential protective factors for infant and young children’s mental health. A secure attachment relationship allows the infant’s developing brain to develop capacities in building and maintaining relationships, emotional regulation, attention and self-control and sets a strong foundation for the later development of resilience, confidence and adaptability (Benoit, 2004; Balbernie, 2013). Researchers have consistently found that securely attached children experience stronger relationships with their parents as well as enhanced problem solving abilities, improved peer relationships and longer lasting friendships (Schneider et al., 2001; Abraham and Kerns, 2013; Guild et al., 2017). These children may also have better sibling relationships, more positive self-esteem, an increased sense of hopefulness, greater trust in people and relationships, and heightened optimism about their future compared to children with insecure attachment styles. In contrast, insecure and disorganised attachment styles in infancy have been associated with elevated rates of emotional, social and behavioral disturbances in infancy, toddlerhood, preschool and beyond (Van Ijzendoorn et al., 1999; Granot and Mayseless, 2001; Sroufe, 2005; Berlin, 2008; Fearon et al., 2010; Madigan et al., 2013). A 30-year prospective study of infants with insecure attachment styles at 8 months of age, found insecure attachment to be associated with a higher risk of mental health concerns at 30 years of age (Fan et al., 2014). Disorganised attachment in infancy is associated with the highest risk of later social and cognitive difficulties and psychopathology with an association found between disorganised infant attachment and childhood behavior problems (Van Ijzendoorn et al., 1999), externalizing and internalizing problems in early school years, aggression and oppositional defiant disorder (Green and Goldwyn, 2002; Fearon et al., 2010), and personality disorder (Steele and Siever, 2010). Studies have found disorganised attachment is significantly correlated with psychopathology in adolescence (Carlson et al., 1998), borderline personality disorder symptoms in adulthood (Carlson et al., 2009), dissociation (Lyons-Ruth, 2003); and post-traumatic stress disorder (PTSD) (Macdonald et al., 2008).

Ideally, parents and caregivers are able to tune into their baby’s cues, interpret their meaning, and respond to them in a contingent, consistent and competent way, which has been termed sensitive parenting (Petch et al., 2012; Ensink et al., 2016). Parents who provide care in this way allow their infant to develop optimal early social-emotional skills, secure infant-parent relationships and cognitive ability (The National Health and Medical Research Council, 2017). One way to understand the connection between sensitive parenting, attachment and later functioning is through the concept of mentalization. Fonagy and colleagues drew on the fields of psychoanalytic theory, attachment theory, theory of mind and developmental psychology to develop the concepts of mentalization theory (Fonagy et al., 1991, 2002; Fonagy and Target, 1997). They defined mentalizing capacity as the ability to understand that that one’s own behavior and the behavior of others is driven by internal states, such as intentions, thoughts, desires, feelings, beliefs, goals and motivations. They proposed that mentalizing capacity develops within the context of a secure attachment relationship (Fonagy et al., 2002), and is key to understanding the association between insecure attachment and psychopathology. Parental mentalizing has been operationalized as parental Reflective Functioning (PRF), and refers to the quality of mentalizing in the context of attachment relationships, and the parent’s capacity to think about mental states in relation to their own and their child’s behavior. Parental RF is considered to play an important role in parenting, and therefore the development of children’s attachment security (Stacks et al., 2014; Ensink et al., 2016; Camoirano, 2017; Barlow et al., 2021). Parents with higher PRF display more sensitivity in their interactions with their infants, and are more likely to have securely attached infants (Grienenberger et al., 2005; Slade et al., 2005a; Rosenblum et al., 2008). Parents with low PRF are more likely to display insensitive parenting and to have to have children with insecure or disorganized attachment styles (Grienenberger et al., 2005; Slade et al., 2005a; Suchman et al., 2010; Stacks et al., 2014; Ensink et al., 2015, 2019).

Only a few studies have been conducted to investigate the connection between parental RF and early childhood psychopathology. These studies have investigated the role of parental RF on the development of child emotional regulation and anxiety symptoms (Camoirano, 2017). Esbjørn et al. (2013) found that low maternal RF predicted higher levels of anxiety amongst clinically anxious school aged children. Heron-Delaney et al. (2016) found that preterm infants with mothers with high RF demonstrated the most negative effects and better self-soothing behavior during the still face procedure compared to infants with mothers with low RF. They surmised that high maternal RF promotes emotional regulation in the infant when distressed, and higher trust in maternal responsiveness. Smaling et al. (2016) found that high risk young pregnant women with higher RF reported significantly lower aggressive behaviors in their children when they were 6, 2, and 20 months old. Ensink et al. (2016) found that maternal RF was found to correlate with child reflective functioning, and negatively with child externalizing behaviors. These findings suggest that maternal mentalizing capacity plays an important role in promoting infant and child emotional regulation, especially in the context of difficult early childhood experiences (Camoirano, 2017). Consequently, enhancing parental mentalizing capacity and parent child attachment security will promote positive mental health in infants and young children, and may work toward preventing mental health difficulties.

To date, most research in this field has focused mainly on the role of mothers. Evidence suggests that mothers and fathers play different and unique roles roles as attachment figures and in socialization and emotion regulation (Benbassat and Priel, 2015; Buttitta et al., 2019). Research is expanding to consider the impact of paternal reflective functioning on child development (Sarkadi et al., 2008; Benbassat and Priel, 2015; Cooke et al., 2017).

## Co-Parenting and the Couple Relationship

In addition to the development of mental health difficulties, many couples also experience a sharp decline in their couple relationship during the transition to parenthood (Lawrence et al., 2008; Doss et al., 2009). In fact, a decline in relationship satisfaction can act as a risk factor for the development of mental health difficulties (Lancaster et al., 2010; Whisman et al., 2011; Giallo et al., 2013; Bayrampour et al., 2015). Meanwhile the inverse may also be true, wherein mental health difficulties may act as a contributing factor for relationship decline (Whisman et al., 2011; Trillingsgaard et al., 2014). Furthermore, researchers have shown that a strong couple relationship can act as a protective factor against the development of perinatal depression and anxiety in both parents (Banker and LaCoursiere, 2014; Pilkington et al., 2015). The couple relationship also has strong links to child outcomes (Cowan and Cowan, 2002; Harold and Leve, 2012). For example, relationship discord in parents has been associated with, poor child adjustment (Hanington et al., 2012), anxiety and depression (Yap et al., 2014), aggression (Cowan and Cowan, 2002), poor academic attainment (Harold et al., 2007) and behavioral issues (Linville et al., 2010). Having a positive couple relationship has also been linked to more responsive parenting (Ponnet et al., 2013). These findings reinforce the importance of the couple relationship during the transition to parenthood.

However, another body of research has gone on to highlight the importance of an additional related, but distinct aspect of the parental relationship, known as coparenting (Feinberg, 2002). Coparenting is defined as the degree to which parents are able to work together harmoniously for the wellbeing of their children (Le et al., 2016). As with relationship satisfaction, coparenting has been linked to a variety of child outcomes (Le et al., 2016), including psychological adjustment (Teubert and Pinquart, 2010), attention and educational achievement (Dopkins Stright and Neitzel, 2003) along with the development of receptive language and social relationships (Cheng et al., 2009). Coparenting has also been related to parenting practices including sensitivity and warmth within parent-child interactions (Cabrera et al., 2009). In fact, some research suggests that coparenting may act as a mediating factor in the link between relationship satisfaction and positive parenting practices (Bonds and Gondoli, 2007; Pedro et al., 2012). In line with this, a large body of research has found a link between relationship satisfaction and positive coparenting (Christopher et al., 2015; Le et al., 2016; Durtschi et al., 2017). Furthermore, in addition to links between coparenting, relationship satisfaction and child outcomes, Feinberg and colleagues have also shown that targeting the coparenting relationship can have a positive impact on parental mental health (Feinberg and Kan, 2008; Feinberg et al., 2016). This research overall demonstrates the importance of coparenting and the couple relationship for both parent and child wellbeing during the transition to parenthood.

## Need for Prevention and Early Intervention

This body of literature highlights the importance of childhood experiences across the lifespan, starting in the perinatal period, emphasizing the importance of addressing risk factors early in life (Felitti et al., 1998; Jones et al., 2018b). The cost of mental health disorders on individuals and society demands a response that focuses on early investment, health promotion and early intervention in an effort to positively impact future health (Jenkins et al., 2002). The World Health Organization has asserted that prevention is the only sustainable approach for reducing the burden of illness associated with mental disorders (World Health Organization, 2004). It is well established that early detection, assessment and intervention of mental health problems in infancy and early childhood is more successful and cost effective than treatment when symptoms become more severe (Davis et al., 2010; Huberty, 2012; The National Health and Medical Research Council, 2017).

This shift in focus toward the prevention of mental illness means that we must consider the mental health and wellbeing of infants and young children, as well as their parents (Guy et al., 2016). While the period of infancy and early childhood is a time when mental health difficulties can develop, it is also an enormously influential developmental stage with the potential to modify or prevent the development of dysfunctional pathways (Karevold et al., 2009; Lewis et al., 2014; Moore et al., 2017). Because many disorders can be prevented through developmentally appropriate, high quality programs and services, it is becoming increasingly acknowledged that it is not enough to merely treat mental health disorders as they emerge (Andrews and Wilkinson, 2002; Waddell et al., 2007). Instead, research suggests that efforts should focus on the prevention of mental health difficulties before they arise, particularly during the earliest stages of life when there is the greatest capacity to effect change (Maldonado-Duran et al., 2000; Bayer et al., 2010b; The National Health and Medical Research Council, 2017).

Focused interventions early in life are one effective method to address these risk factors and reduce and prevent poor outcomes in infants and young children before they emerge, allowing for better outcomes later in life. An increased emphasis on the importance of the perinatal and infancy period has contributed to the development of programs that aim to either prevent the emergence of mental health disorders or intervene early if they do develop (Van Ravesteyn et al., 2017). Prevention refers to any approach that is applied in an effort to prevent later difficulties and to enhance the cognitive, behavioral, emotional, social and physical development of young children during the period from pregnancy to 6 years of age (Zero to Three, 2012; Dunst et al., 2014). Prevention programs are often offered on a community wide basis, especially in high-risk communities; they serve to intervene early and support parents and caregivers to provide sensitive, warm and secure relationships and detect emotional problems before they become more resistant to change (Mihelic et al., 2017).

Early intervention refers to interventions offered once an infant/child or their parents are identified as significantly at risk or the child is already showing some type of difficulty that is seen as placing their development at risk (Dunst et al., 2014). Over the past decade, several perinatal and infancy prevention/early intervention programs have been developed and evaluated, with some designed for universal implementation and others designed to be implemented in communities who are at increased risk of poor social and emotional development. These programs aim to improve mental health in both parents and children, and are a window of opportunity through which to enhance the social and emotional development of infants and young children (Watson et al., 2005). In this article, we look at the existing programs that have been developed as interventions during the early years of life. This is important to identify which programs and approaches are most effective, and to build on the research that has been conducted over the past years.

## Method

This review aimed to look at (1) the existing programs targeting mental health in children aged 0–3 and their parents (2) the components and efficacy of these programs.

A search was conducted across multiple online databases, including PsychInfo, Informit, Scopus, ProQuest, Wiley Online Library, Science Direct, PubMed and Google Scholar. Reference tracking of relevant articles was also used, and websites of specific programs were searched for reference lists. The search terms used in the databases included: perinatal mental health, infant mental health, early life programs, parenting interventions, prevention, anxiety, depression, coparenting, reflective functioning, mentalization, attachment. An initial search using these terms generated 10,809 peer reviewed articles. These were further narrowed down according to inclusion and exclusion criteria, through specification of search terms and visual inspections of titles and abstracts.

Articles were included if they were: written in English, detailed an intervention or program for children aged 0–3 years, and were focused on the mental health of parents or children. Articles were excluded if they were: not in English, did not contain an intervention or program, involved programs directed at older children, or involved programs solely focused on medical or physical aspects of infant health, e.g., premature birth. The search was concluded on the 20th March, 2020, resulting in 60 articles reporting 27 interventions that were relevant to the current review. Of the 60 articles, 9 were descriptive only, leaving 51 articles that reported original data and results of programs.

In this review, we have included effect sizes for programs where they were reported or where there was enough information reported to calculate them manually (Lakens, 2013). Instead of providing descriptions of effect sizes throughout the review, they are detailed below (see Table 1). Details of the included articles are outlined briefly (see Table 2) and then described more extensively.

**TABLE 1:** Effect sizes.

Effect size	Small	Medium	Large
*d*	0.20	0.50	0.80
*r*	0.10	0.30	0.50
η_*p*_^2^ (partial eta squared)	0.01	0.06	0.14
*g*	0.20	0.50	0.80
ES (effect size for one group design)	0.20	0.50	0.80

**TABLE 2:** Articles in review.

Program and author	Participants	Control group	Significant effects and size (S = small, M = medium, L = large, ? = not provided)
**Interventions that target maternal mental health**			
Mindful Motherhood			
Vieten and Astin, 2008	31 mothers, targeted	Waitlist control	Anxiety (L), negative affect (L)
ROSE Program			
Zlotnick et al., 2001	37 mothers, targeted	Treatment as usual	Depressive symptoms (?), risk of developing PND (?)
Zlotnick et al., 2006	99 mothers, targeted	Standard antenatal care	Risk of developing PND (?)
Johnson et al., 2018	N/A	N/A	N/A
**Interventions that target maternal mental health and parenting skills**			
Toward Parenthood			
Milgrom et al., 2011	143 mothers, universal	Routine care	Depression (M), anxiety (M), stress (M), parenting dysfunction (M)
PREPP			
Werner et al., 2016	54 mothers, targeted	Enhanced treatment as usual	Depressive symptoms (L), anxiety symptoms (M)
Mothers and Babies			
Munoz et al., 2007	41 mothers, targeted	Usual medical care	No significant effects
Tandon et al., 2011	61 mothers, targeted	Standard visits plus information	Depressive symptoms (S)
Le et al., 2011	217 mothers, targeted	Usual care	No significant effects
Mendelson et al., 2013	78 mothers, targeted	Standard visits plus information	Mood regulation (?)
Tandon et al., 2014	78 mothers, targeted	Home visiting as usual	Depressive symptoms (L)
Leis et al., 2015	15 mothers, targeted	No control group	Depressive symptoms (L), mood regulation (L
McFarlane et al., 2017	95 mothers, targeted	Home visiting as usual	Depressive symptoms (S, stress (S)
Tandon et al., 2018	120 mothers, universal	Home visiting as usual	Depression (M), anxiety (S)
CAPEDP			
Saias et al., 2013	N/A	N/A	N/A
Dugravier et al., 2013	440 mothers, targeted	Usual care	Depression for low-risk mothers (?)
Antenatal group program			
Thomas et al., 2014	48 mothers, targeted	No control group	Depression (L), anxiety (L), maternal attachment (M)
**Interventions that target fathers**			
Boot Camp for New Dads			
Capuozzo et al., 2010	N/A	N/A	N/A
Miller, 2012	2301 fathers, universal	No control group	Parenting confidence (?)
**Interventions that target the couple relationship**			
Couple CARE for Parents			
Halford et al., 2010	71 couples, universal	Maternal parenting program	Couple conflict (L), invalidation (L), negative affect (L), women’s relationship adjustment (M), self-regulation (L)
Petch et al., 2012	250 couples, targeted	Maternal parenting program	Conflict for women (M), invalidation for women (L)
Heyman et al., 2019	368 couples, targeted	Waitlist control	No significant effects
**Interventions that target the mother-infant relationship**			
AMPLE			
Nicolson et al., 2013	97 mothers, targeted	Usual care	Maternal non-intrusiveness (L), maternal non-hostility (M)
Mom Power			
Muzik et al., 2015	99 mothers, targeted	No control group	Depressive symptoms (?), PTSD symptoms (?), caregiving helplessness (?), parenting reflectivity (?)
Muzik et al., 2016	49 mothers, targeted	No control group	Depressive symptoms (?), PTSD symptoms (?)
Rosenblum et al., 2017	122 mothers, targeted	Mailout information	PTSD symptoms (S), parenting stress (S), depressive symptoms [iatrogenic] (S), depressive symptoms in women with IPV history (M), PTSD symptoms in women with IPV history (M)
Rosenblum et al., 2018	75 mothers, targeted	Mailout information	Reflective parenting (S)
Playing and Learning Strategies			
Landry et al., 2006	242 mothers, targeted	Developmental assessment sessions	Contingent responsiveness (L), support of infant foci of attention (M), verbal scaffolding (L), object labeling (L), smiling and laughing [iatrogenic] (M)
Landry et al., 2008	166 mothers, targeted	Developmental assessment sessions	Verbal encouragement (S), child cooperation (S), social engagement (S), contingent responsiveness (M), negative behavior (S)
Attachment skills program			
Akbarzadeh et al., 2016	190 mothers, universal	Routine care	Anxiety (?), infant crying (?)
HUGS			
Milgrom et al., 2006	22 mothers, targeted	No control group	Parenting stress (?)
Milgrom and Holt, 2014	100 mothers, targeted	Attention placebo	N/A
Holt et al., 2021	77 mothers, targeted	Attention placebo	Affection involvement and verbalisation (L), bonding (M)
**Parental Reflective Functioning based interventions**			
Families First			
Kalland et al., 2016	N/A	N/A	N/A
PEEP Reflective Parenting Program			
Maskell-Graham, 2014	10 mothers, targeted	No control group	Reflective functioning (?), mother-baby interaction (?)
**Lighthouse Parenting Program**			
Byrne et al., 2018	16 parents, targeted	No control group	Parental self-efficacy (L)
Circle of Security			
Hoffman et al., 2006	65 mother-infant dyads, universal	No control group	Attachment style (?)
Cassidy et al., 2010	20 mother-infant dyads, targeted	No control group	Depressive symptoms (L)
Cassidy et al., 2011	220 mother-infant dyads, targeted	Psychoeducation sessions	Attachment style among highly irritable infants (?)
Kohlhoff et al., 2016	15 mother-infant dyads, universal	No control group	Reflective functioning (M), caregiving helplessness (L), maternal rejection and anger (M), maternal stress (M)
Yaholkoski et al., 2016	10 studies, universal and targeted	Meta-analysis, various control groups	Child attachment security (M), caregiving quality (M)
Rose et al., 2018	9 parents, universal	No control group	Self-efficacy (M)
Mothander et al., 2018	52 parent-infant dyads, targeted	Treatment as usual	Caregiver perceptions (?), emotionally available interactions (?)
Minding the Baby			
Slade et al., 2005b	N/A	N/A	N/A
Sadler et al., 2013	105 mothers, targeted	Routine care	Infant attachment (?)
Slade et al., 2020	164 mothers, targeted	Treatment as usual	Maternal reflective functioning (?), infant attachment (?)
**Interventions targeting child wellbeing**			
Families in Mind			
Bayer et al., 2010a	733 families, universal	Usual primary care	Maternal unreasonable expectations (?)
Hiscock et al., 2012	N/A	N/A	N/A
Tuning in to Toddlers			
Lauw et al., 2014	34 mother-toddler dyads, universal	No control group	Emotion coaching beliefs (M), emotion coaching behaviors (L), observed emotion coaching (L), emotion labels (L), emotion exploration (L), emotion-dismissing beliefs (L), emotion-dismissing behaviors (L), observed emotion dismissing (M), toddler behavior problems (S)
Havighurst et al., 2019	N/A	N/A	N/A
Relationships for Growth and Learning			
Bekar et al., 2017	47 children, targeted	No control group	Social competence (?), behavioral problems (?)
Early Head Start			
Administration for Children, Youth and Families, 2001	3000 families, targeted	Access to other services	Cognitive and language development (?), problem behaviors (?), parent support (?), family support (?), parenting stress (?)
Raikes and Love, 2002	N/A	N/A	N/A
Love et al., 2005	3001 families, targeted	Access to other services	Cognitive and language functioning (S), aggressive behaviors (S), emotional engagement (S), sustained attention (S), parent-child interactions (S)
Responsive Early Childhood Curriculum			
Landry et al., 2014	542 children, targeted	Business as usual	Expressive emotion understanding (M), receptive emotion understanding (S), situational emotions task (M), social competence (M), anger and aggression (M)
**Interventions targeting parent mental health, the couple relationship and child wellbeing**			
Family Foundations			
Feinberg and Kan, 2008	169 couples, universal	No-treatment control	Coparental support (M), parenting-based closeness (M), maternal depression (M), maternal anxiety (M), parent-child dysfunctional interaction (L), infant duration of orienting (S)
Feinberg et al., 2014	169 couples, universal	Sent information in post	Child internalizing problems (M), internalizing problems for boys (L)
Jones et al., 2018a	399 couples, universal	Sent information in post	Triadic relationship quality (M), negative coparenting (M), negative parenting (M), child internalizing problems (S), child night wakings (S)
Baby Triple P			
Tsivos et al., 2015	27 mothers, targeted	Treatment as usual	No significant effects
Popp et al., 2019	49 couples, universal	Care as usual	Infants awake and content (L), inconsolable crying (L)
What Were We Thinking			
Fisher et al., 2010	399 couples, universal	Usual primary health care	Risk of depression, anxiety or adjustment disorder for mothers with a psychiatric history (?)
Fisher et al., 2016	400 couples, universal	Usual care	Self-rated health (?)

## Results

Due to the young age of the children concerned in this review, the majority of the programs (24) found in our search were designed to be presented to parents, addressing parental factors that would impact their infants and toddlers. Only a few (3) of the programs were designed to be presented specifically to children (*Relationships for Growth and Learning*, *Early Head Start*, and *Responsive Early Childhood Curriculum*).

The highest proportion of existing programs were targeted interventions for at-risk mothers (*PREPP*, *ROSE Program*, *PEEP Reflective Parenting Program*, *Lighthouse Parenting Program*, *Minding the Baby*, *Mindful Motherhood*, *AMPLE*, *CAPEDP*, *Mom Power*, *Playing and Learning Strategies*, *HUGS* and an unnamed antenatal group program). Across programs, a number of different maternal risk factors were used as inclusion criteria for participation in the interventions, which included: symptoms of anxiety and depression, low income or socioeconomic status, risk of intimate partner violence, a history of trauma, being in a psychologically vulnerable situation, being an adolescent or young mother, being in jail and having relationship problems.

The existing interventions also included universal programs (*Family Foundations*, *Towards Parenthood*, *What Were We Thinking*, *Families First*, *Boot Camp for New Dads*, *Families in Mind*, *Tuning into Toddlers* and an unnamed attachment skills program) and programs that could be delivered as either targeted or universal (*Baby Triple P*, *Couple CARE for Parents*, *Mothers and Babies* and *Circle of Security*). Some of the programs were specifically designed for use with couples (*Family Foundations*, *What Were We Thinking* and *Couple CARE for Parents*) while only one of the programs was developed especially for fathers (*Boot Camp for New Dads*). Many of the programs enabled partners to be involved, but did not have this as a main focus of the intervention. We present an overview of each of the programs below.

## Existing Early Life Interventions for Parents

### Interventions That Target Maternal Mental Health

The *Mindful Motherhood* intervention uses aspects of mindfulness based cognitive therapy and acceptance and commitment therapy, with the goal of reducing the risk of adverse maternal mental health outcomes during the perinatal period (Vieten and Astin, 2008). The program is targeted for women who have had low mood during pregnancy. The content is presented in group settings; a clinical psychologist and yoga instructor jointly facilitate these 2 h sessions once weekly for 8 weeks. The sessions include discussions, provision of information and activities, such as body scan exercises and mindful movement. The mothers are also given guided meditations that they can do every day at home (Vieten and Astin, 2008).

The intervention women showed significant decreases in anxiety (*d* = 0.89) and negative affect (*d* = 0.83) compared to control group women. Improvements were also observed in depression, positive affect, mindfulness, affect regulation and stress; however, these changes were not significant (Vieten and Astin, 2008).

The *ROSE Program* (Zlotnick et al., 2001) is targeted for women who are on public assistance and at risk of postnatal depression. It uses interpersonal therapy to support the transition to motherhood and build support networks. The program includes content on managing the role transition to becoming a mother, goal setting, interpersonal conflict resolution and developing social supports. The content is presented through four 1-h long group sessions, run weekly.

A pilot study of the program found that intervention group women displayed a significantly greater improvement in depression symptoms as well as being at lower risk of developing postnatal depression (Zlotnick et al., 2001). A larger study found that women who participated in the program were significantly less likely to develop postnatal depression compared to those who did not (Zlotnick et al., 2006). Effect sizes were not provided in these papers. An additional study is currently underway (Johnson et al., 2018).

### Interventions That Target Maternal Mental Health and Parenting Skills

*Towards Parenthood* is a program for new mothers that targets risk and protective factors for postnatal depression, anxiety and parenting difficulties (Milgrom et al., 2011). There are two parts to the program: a nine unit self-help workbook with telephone support and a community networking component. Eight of the units are antenatal and one is postnatal. The units include: cognitive behavioral strategies, problem solving, reflecting on own life events, building self-esteem, parenting skills and infant bonding. The program addresses partner communication and finding a wider support network. Partners were also able to be involved in the units.

The efficacy of the program has been tested, indicating that the intervention was associated with lower levels of depression (*d* = 0.6), anxiety (*d* = 0.58) and stress (*d* = 0.59) symptoms for mothers. Parenting dysfunction was also lower in the intervention group (*d* = 0.46). Partners in the intervention group also scored lower in depression and anxiety, however, the significance could not be determined due to low numbers of partners who completed the measures.

*Practical Resources for Effective Postpartum Parenting (PREPP)* is a targeted intervention for women at risk of postnatal depression; it is also focused on optimizing infant behavioral outcomes (Werner et al., 2016). The intervention uses traditional psychotherapy, psychoeducation and mindfulness techniques. It also builds caregiving skills, teaching mothers how to soothe their babies and increase infant sleep using five specific behavioral techniques. The intervention focuses on the mother-infant dyad. Mothers receive three individual sessions with a psychologist, two before birth and one after.

A study found that the intervention resulted in significant reductions in symptoms of depression (*d* = 0.815) and anxiety (*d* = 0.664), as well as fewer fussing and crying behaviors for babies at 6-weeks postpartum. However, these effects were not sustained at 10 and 16 weeks.

*Mothers and Babies* is a home visit program targeted for pregnant women who meet low income or other at-risk criteria (Tandon et al., 2018). There are 12 sessions in the program that can be delivered as part of an existing home visit schedule. *Mothers and Babies* is based on cognitive behavioral therapy and attachment theory. The main cognitive behavioral topics in the program are pleasant activities, thoughts and contact with others. The content is presented through interactive activities. The program has been widely used and tested in a number of studies, displaying efficacy in improving mental health.

The initial study of the program found a small (*h* = 0.28) but insignificant difference in depressive symptoms favoring the intervention group (Munoz et al., 2007). Other studies have found improvements in depressive symptoms at 3-months (partial eta squared = 0.01) and 6-months (*d* = 0.73) follow-up (Tandon et al., 2011, 2014). Another study found significant improvements in mood regulation as a result of the program (Mendelson et al., 2013).

A study targeting low income mothers found a post intervention decrease in depressive symptoms (*d* = 0.38) and stress (*d* = 0.35) that was greater than that of the control group; however, this was not maintained at 6-months follow up (McFarlane et al., 2017). The most recent study testing an adapted version of the program (Tandon et al., 2018) found significant effects on depression (*d* = 0.444) and anxiety (*d* = 0.175) at 6-months follow-up for intervention group mothers. Effect sizes were not included but were calculated from the information provided.

The program has also been run in a clinic as opposed to a home visiting format (Leis et al., 2015). A pilot study using this format with an intervention only group found significant improvements in depressive symptoms (*ES* = 0.78) and mood regulation (*ES* = 0.84) after the intervention. Another study using the group clinic format found that levels of depression did not differ between the intervention and control groups (Le et al., 2011).

The *CAPEDP* intervention is a home visiting program that targets the mental health of infants within families living in vulnerable situations (Saias et al., 2013). Other target outcomes include reduced maternal depression and improved family environment. The program is extensive, involving 44 home visits beginning in the third trimester and ending around the child’s second birthday. Trained psychologists conduct the visits, which are all manualised. The intervention uses video footage of the mother and her baby, which is discussed with the psychologist. Other features include short films on maternity topics, information on community support services, and assistance in developing parenting skills (Dugravier et al., 2013; Saias et al., 2013).

A randomized controlled trial of the program did not find an overall effect on maternal depressive symptoms, but did find effects on subgroups within the study (Dugravier et al., 2013). The intervention was able to reduce depression for women who had lower baseline levels of depression, were planning to bring up their child with the child’s father and who had completed more than 9 years of education. There was not enough data reported to calculate effect sizes.

*An antenatal group program* was developed focusing on behavioral self-care strategies, psychoeducation for managing mental health, interpersonal therapy and the parent-infant relationship (Thomas et al., 2014). It is targeted for women who have current symptoms of depression or anxiety or are at-risk due to other factors. Women attend six sessions of the program, with a 2-h session each fortnight. Partners are able to attend two of these sessions. The delivery of the program is based on elements of cognitive-behavioral therapy and parent-infant interventions. Mothers and their partners are provided with information and strategies, covering topics such as bonding with infants, responsive parenting, engaging in self-care, contingency planning for emerging mental health problems, and recognizing mental health warning signs in each other (Thomas et al., 2014).

In a targeted pilot study, women who completed the program displayed significant decreases in depression (*d* = 1.1) and anxiety (*d* = 0.7), as well as increases in maternal attachment (*d* = 0.5). Of partners who attended the sessions, 81% indicated that their understanding of mental health issues had improved (Thomas et al., 2014).

### Interventions That Target Fathers

*Boot Camp for New Dads* (Capuozzo et al., 2010) appears to be one of the only interventions targeted specifically for new fathers. Fathers learn how to prepare for their baby, support their partner and bond with their baby. The program is run in groups, with both “rookie” fathers and “veteran” fathers, as well as a group coach. “Rookie” fathers attend one session before birth and are invited back after birth to share their new experiences as “veteran” fathers.

The majority of fathers who participated in the program reported increased confidence across multiple areas (Miller, 2012), such as: caring for their baby (92%), dealing with crying (90%), bonding (78%), understanding their partner’s emotions (89%), supporting their partner (89%), protecting their family from negative outside influences (84%), creating a safe environment for their baby (77%), developing their own style with the baby (82%) and forming a parenting team with their partner (89%). Qualitative research has also been conducted; however, no randomized controlled trials have been run to test the program’s outcomes (Miller, 2012).

### Interventions That Target the Couple Relationship Only

*Couple CARE for Parents* (Petch et al., 2012) focuses on preparing couples for the transition to parenthood by enhancing their couple relationship and building mutual support. It aims to promote communication between parents, especially with regards to parenting. The program includes content on mental health and self-care, such as: education about how to prevent, detect and seek help for perinatal depression and anxiety, mindfulness, breaking the spiral of depression, CBT and self-monitoring. The program is delivered over six units – one group session, two home visits and three phone calls. Trained facilitators walk through the program with couples and guide them through the various topics.

The program has been able to decrease negative couple communication at post-test, specifically conflict (females, *r* = 0.84; males, *r* = 0.92), invalidation (females, *r* = 0.82; males, *r* = 0.86) and negative affect (females, *r* = 0.84; males, *r* = 0.92), these being large effects (Halford et al., 2010). Smaller effects were found for women’s relationship adjustment (*r* = 0.32) and self-regulation (*r* = 0.46) at 12-months postpartum. Effects were not found for parenting stress. In another study, the program reduced conflict (*d* = 0.38) and invalidation (*d* = 0.44) in women (Petch et al., 2012). It also prevented deterioration in relationship satisfaction for high risk women but displayed no effects for men. The program did not appear to have an impact on intimate partner violence or relationship problems (Heyman et al., 2019).

### Interventions That Target the Mother-Infant Relationship

The *Adolescent Mothers’ Program: Let’s meet your baby as a person* is a targeted program for adolescents mothers (AMPLE; Nicolson et al., 2013). The intervention focuses on the mother-infant relationship and is run across two sessions, in addition to regular maternity care. The first of these is a group session where mothers watch and discuss video clips about connecting with their newborn. The second session is individual, and each mother talks through getting to know her own baby. Although the main focus is building attachment between the mother and baby, partners are also able to attend.

A study found significant effects on some aspects of emotional availability, including maternal non-intrusiveness (*d* = 1.06) and maternal non-hostility (*d* = 0.66) in a play only task and maternal non-intrusiveness (*d* = 0.85) and maternal non-hostility (*d* = 0.66) in a play plus separation-reunion task (Nicolson et al., 2013). Effects were non-significant for maternal sensitivity and structuring, or child responsiveness and involvement.

The *Mom Power* program aims to build secure attachment between mothers and their babies, using a delivery method of psychoeducation and skills training (Muzik et al., 2016). The program is particularly designed for mothers who have previously experienced or are currently experiencing trauma (Rosenblum et al., 2018). A major goal of the intervention is to encourage sensitive parenting through skills practice and reflection. Mothers are also provided with opportunities to acquire self-care skills and social support. The intervention consists of 10 group sessions and 3 individual sessions, led by two trained facilitators. Each group session, to which mothers can bring all of their children less than 6 years old, begins with a shared meal. During the rest of the session, mothers work through the manualised program, while children take part in a separate play-based session (Muzik et al., 2015). To complete the session, mothers and children take part in a fun activity together.

Intervention only studies have been conducted, comparing completers and non-completers of the program (Muzik et al., 2015, 2016). Results indicate that the program is able to contribute to decreases in depressive symptoms, PTSD symptoms, maternal helplessness and clinical diagnoses (Muzik et al., 2015, 2016). As these studies did not have control groups, subsequent studies used randomized controlled trials to test for efficacy.

A study involving high-risk mothers (Rosenblum et al., 2017) found that women in the intervention group decreased significantly in PTSD symptoms (*d* = 0.342) and parenting stress (*d* = 0.334); however, the opposite effect was seen in that control group women decreased significantly in depressive symptoms, while the intervention group did not (*d* = 0.233). Among women with a history of intimate partner violence, strong effects were found for the intervention group in decreased symptoms of depression (*d* = 0.462) and PTSD (*d* = 0.401). Another study found an increase in reflective parenting (*d* = 0.296) for the intervention but not the control group (Rosenblum et al., 2018).

*Playing and Learning Strategies* (PALS) is a ten-week home visiting program that targets maternal responsiveness in order to enhance infant outcomes (Landry et al., 2006). It is targeted especially for mothers from a low income background or for infants with low birth weight, and emphasizes taking the family context into account. Reflecting on videotaped mother-baby interactions is a major component of the intervention. Each week, the facilitator coaches the mother through a new target behavior, and films the mother interacting with her baby. They also spend time discussing the videotaped interaction from the previous week, and plan how to incorporate the target behaviors into daily life. Mothers learn how to observe the responses of their infants. The toddler program (Landry et al., 2008) builds upon the infant program, continuing to teach mothers responsive behaviors through coaching and reflection on videotaped interactions.

The program has demonstrated increases in aspects of maternal responsive behaviors (Landry et al., 2006), including contingent responsiveness (*d* = 0.85), support of infant foci of attention (*d* = 0.65), verbal scaffolding (*d* = 0.79) and object labeling (*d* = 0.71). However, an iatrogenic effect was found among high-risk mothers, with the control group mothers displaying higher levels of smiling and laughing than intervention group mothers (*d* = 0.55). Effects were also seen in infants, including significantly increased early communication (*d* = 0.75) and social cooperation (*d* = 0.50) with their mothers. A subsequent study (Landry et al., 2008) examined the effects of further intervention at the toddler stage. Intervention at the toddler stage was linked to significant effects on verbal encouragement (*d* = 0.25), child cooperation (*d* = 0.30) and child social engagement (*d* = 0.32). Mothers and toddlers who participated in the intervention at both stages had higher levels of contingent responsiveness (*d* = 0.51) and more decreases in negative behavior (*d* = 0.16).

*A universal attachment skills program* was developed to target infant mental health through building mother-infant attachment (Akbarzadeh et al., 2016). The program consists of four sessions, run once weekly for 60–90 min, with session content delivered through discussions, time for questions, lecture-style provision of information, videos and role-plays. The sessions have a large focus on attachment behaviors, with mothers also learning about the physiology of pregnancy, awareness of their embryo’s feelings, and maintaining good health during pregnancy.

At the time of birth, mothers in the control group scored significantly higher in anxiety, while babies in the intervention group had significantly less crying. However, baseline data for mothers was not collected prior to the intervention being run (Akbarzadeh et al., 2016).

*HUGS* (Milgrom and Holt, 2014) is an intervention targeted for women with a diagnosis of postnatal depression. The four week program includes education and activities that address mother-infant interaction; mothers are given opportunities to interact with their infants while learning practical skills such as recognizing infant cues. The HUGS program is designed to be run in conjunction with cognitive behavioral therapy for postnatal depression.

A pilot study of a previous three week version of the program found a decrease in parenting stress among women who participated. However, there was no control group. A subsequent study combining the HUGS intervention with a nine week cognitive behavioral program explored the effects on the mother-infant relationship, maternal mood and infant wellbeing (Milgrom and Holt, 2014). Preliminary results indicate improvements in affective involvement and verbalisation (partial eta squared = 0.10) and bonding (partial eta squared = 0.09) for the intervention group mothers. Results were not found for parenting stress or infant outcomes (Holt et al., 2021).

### Mentalization Based Interventions That Target Parental Reflective Functioning

*Families First* is a parenting group program that has a unique focus on parental reflective functioning (Kalland et al., 2016). It aims to improve parent-child attachment through building mentalization capacity. It focuses on the whole family and also teaches parents to develop a wider social support network. The program was designed to reduce parenting stress and depression. The groups are offered for first time parents whose babies are 3–4 months old. There are twelve sessions in the program, with each running for approximately 2 h. To date, there have been no results published on the efficacy of the program.

The *PEEP Reflective Parenting Program* is designed for expectant parents and is delivered during the perinatal period (Maskell-Graham, 2014). The program aims to build parental reflective functioning capacity and develop secure attachments between parents and babies. Parents are encouraged to view their babies as individuals with their own internal thoughts and feelings. Another goal of the program is to build support networks for parents within the community. The content is delivered through a home visit and seven group sessions. Preliminary results indicated improvements in reflective functioning and mother-baby interaction, but not in anxiety and parenting stress. However, the data was not analyzed statistically so the significance and size of effects could not be determined (Maskell-Graham, 2014).

The *Lighthouse Parenting Program* is a 20-week Mentalization-Based Treatment (MBT) designed for high-risk parents of infants aged 0–2 years (Byrne et al., 2018). This intervention is designed to increase sensitive parenting and prevent child maltreatment through increasing parents’ capacity to mentalize their child’s internal states. The program involves weekly group sessions and fortnightly individual sessions for parents; children are not included in the intervention.

The program displayed a significant effect on parental self-efficacy (*d* = 1.675) for mothers. Non-significant improvements were seen in parental stress, parental sensitivity, anxiety, depression, parental mentalizing and global distress. The low levels of depression and anxiety reported at baseline, as well as the small sample size could have impacted the significance of findings.

The *Circle of Security* (COS) intervention aims to increase parental sensitivity in order to promote the development of secure attachment in children (Kohlhoff et al., 2016). Initially, COS was designed as a 20-session weekly group-intervention intended to enhance parental ability to interpret child cues (Yaholkoski et al., 2016), however, a shorter 8-session version (COS-P) has also been created, with the same key components and aims as the original version (Cooper et al., 2009). Both the original and the shortened versions include an overview of attachment theory as well as videos designed to demonstrate secure and problematic parent-child interactions; however, unlike COS-P, where pre-recorded footage is used, the original version includes videos of participants and their own children filmed in the strange situation (Kohlhoff et al., 2016).

The COS program has been researched extensively since its development. A study involving women in a jail diversion program found that depressive symptoms were significantly lower after the intervention (*ES* = 0.88), yet the rates of attachment security and maternal sensitivity did not differ from those in the population (Cassidy et al., 2010). Another study found significant decreases in disorganized and insecure attachment (Hoffman et al., 2006). A pilot version of the shorter version of the program found favorable effects on reflective functioning (*ES* = 0.59), caregiving helplessness (*ES* = 0.73), maternal rejection and anger (*ES* = 0.48), and maternal stress (*ES* = 0.48) (Kohlhoff et al., 2016). Effects have also been found on aspects of self-efficacy (*ES* = 0.55) (Rose et al., 2018). However, all of these studies used a one-group pretest posttest design and thus did not have a control group, so it is difficult to determine whether or not these findings reflect intervention effects.

The program has also been studied using randomized controlled trials. In a targeted study for economically stressed mothers, effects were not found for the overall sample, however, among highly irritable infants, intervention group children were more likely to be securely attached after the intervention, as compared to the control group (Cassidy et al., 2011). A study of the shortened version found significant increases in balanced caregiver perceptions and emotionally available interactions in the intervention group but not the control group (Mothander et al., 2018).

A meta-analysis has been conducted on the program, although it must be noted that this included data from sources not within the scope of this review, such as unpublished data and articles, and COS as a program for children older than 3 years old (Yaholkoski et al., 2016). The analysis found significant, medium effects on both child attachment security (*g* = 0.65) and caregiving quality (*g* = 0.60).

The *Minding the Baby* (MTB) attachment-based intervention aims to promote secure attachment in infants through increasing maternal reflective functioning in vulnerable young primiparous mothers (Sadler et al., 2013). Beginning in the second trimester of pregnancy, this intensive program endeavors to improve developmental, health and relationship outcomes for participating families (Slade et al., 2020). Families receive weekly alternating visits by a nurse and a social worker for the first year of the child’s life, followed by bi-weekly visits until the child reaches age 2 (Slade et al., 2020). Other family members including the child’s father are encouraged to participate, however, the mother and child remain the focus of this intervention (Slade et al., 2020).

In a study of young mothers, results indicated a significantly higher percentage of secure infant attachment in the intervention group and significantly lower disorganized attachment. There were no significant effects found for maternal mental health or reflective functioning, although there were effects on subgroups within the sample (Sadler et al., 2013). There was a non-significant trend toward less referrals to the Child Protective Services at 24-months follow-up. Another study found significant effects on maternal reflective functioning and infant secure attachment, but not on affective communication or maternal mental health (Slade et al., 2020).

## Existing Early Life Interventions for Children

### Interventions That Target Child Wellbeing

*Families in Mind* (Hiscock et al., 2012) is a combination of two interventions – *Toddlers Without Tears* and *the Family Check-Up.* The *Toddlers Without Tears* program is universal and targets negative parenting styles and child mental health (Bayer et al., 2010a). It is delivered through a combination of one individual session and two group sessions. The parents learn strategies for managing behavior in their toddlers. A study has found that this program alone does not appear to be enough to prevent emotional and behavioral problems in children or mental health problems in mothers. The only effect demonstrated was on mothers’ unreasonable expectations of their toddlers, which were significantly lower in the intervention group (Bayer et al., 2010a). Not enough information was provided to calculate effect sizes. The *Family Check-Up* is targeted and has previously displayed efficacy when delivered to disadvantaged families. In this program, families receive one-on-one home visits from a psychologist. A longitudinal study of the *Families in Mind* program has commenced and is still in progress.

*Tuning into Toddlers* (TOTS) is a universal program designed to enhance emotional development in toddlers through addressing their parents’ emotion socialization and emotion regulation (Havighurst et al., 2019). It is also based on the theories of attachment, mindfulness and neurobiology. In particular, TOTS aims to build emotional coaching skills in parents, as this is a major protective factor for child mental health. The program is run across six weekly sessions, each of which are 2 h long (Lauw et al., 2014). Parents learn the five steps of emotional coaching, for example, being aware of children’s emotions and helping label emotions. They learn information about toddler needs and development. The program is manualised and is presented to groups using discussions, role-plays, psychoeducation and other activities.

A pilot study of the program (Lauw et al., 2014) found a number of effects, including increased emotion coaching beliefs (*d* = 0.50), emotion coaching behaviors (*d* = 1.38), observed emotion coaching (*d* = 0.92), emotion labels (*d* = 0.87) and emotion exploration (*d* = 0.68). There were also significant decreases in emotion-dismissing beliefs (*d* = 0.70), emotion-dismissing behaviors (*d* = 0.80), observed emotion dismissing (*d* = 0.49) and toddler behavior problems (*d* = 0.30). As the pilot study used a small intervention only design, another study using a randomized controlled trial design is currently underway (Havighurst et al., 2019).

The *Relationships for Growth and Learning Program* is designed to target preschool children who are at risk for developing mental health problems (Bekar et al., 2017). The program uses peer play psychotherapy and other mental health services. While the program is essentially child-focused, parents and teachers are also involved, as the program uses a whole system approach. The program was run in childcare centers for children at clinical risk due to internalizing and externalizing symptoms, environmental risk and challenging histories. The children received peer play therapy in groups with a trained therapist. Individual sessions were also given if necessary.

The clinical children increased significantly in social competence and decreased in total problems and behavioral problems. After participation in the program, the clinical group of children had caught up to the nonclinical group of children in behavioral functioning in that they no longer had more total problems, including behavioral, internalizing, externalizing and other problems.

*Early Head Start* (Raikes and Love, 2002) refers to a range of programs for low-income pregnant women and their families, which can be based at the family home or in the community. The programs involves two generations and follows children over the first few years of life.

A large study of the Early Head Start programs, which included data from 3,001 families across 17 programs, measured the efficacy of the programs when children were 2 and 3 years of age (Love et al., 2005). Families within each of the 17 programs were randomly assigned to an intervention or control group. When children were 2 years of age, the results indicated decreased risk for intervention group children in a number of areas, including children’s cognitive and language development (14.9%), problem behaviors (10.2%), parental support (13.5%), parenting stress (11%) and family support in the home environment (11.5%), as compared to the control group children (Administration for Children, Youth and Families, 2001).

At 3 years old, children who were part of the interventions had better cognitive and language functioning (*d* = 0.12), fewer aggressive behaviors (*d* = 0.11) and higher emotional engagement (*d* = 0.20) and sustained attention (*d* = 0.16) with their parents, compared to children in the control group. Parents also displayed better interactions with their children (*d* = 0.11). An analysis of program type revealed that programs that were both home and community based had the most desirable results for children (*d* = 0.23–0.31) and parents (*d* = 0.21–0.28).

The *Responsive Early Childhood Curriculum* (RECC; Landry et al., 2014) was developed for toddlers from low income backgrounds in childcare centers, with the goal of enhancing the quality of care received during the formative years. The program involved equipping teachers with the ability to engage in responsive teacher-child interactions. Teachers were trained and implemented the curriculum in their centers.

A randomized study was conducted, with two intervention groups and one control group. One intervention group used the regular RECC and the enhanced intervention group used the RECC plus extra activities to build social emotional skills. Children in the intervention groups scored higher than control group children in expressive emotion understanding (*d* = 0.47), receptive emotion understanding (*d* = 0.25) and a situational emotions task (*d* = 0.44). Control group children displayed a significant rise in anxiety, but intervention group children did not. Children in the regular (*d* = 0.42) and enhanced (*d* = 0.39) intervention groups increased in social competence at some, but not all time points. Children in the regular intervention group decreased significantly in anger and aggression compared to the control group (*d* = 0.55).

### Interventions That Target Parent Mental Health, the Couple Relationship and Child Wellbeing

*Family Foundations* is a universal program for expectant couples which focuses on enhancing co-parenting and mental health (Feinberg and Kan, 2008). It was developed based on extensive research on the importance of co-parenting, including the dimensions of co-parental support and parenting-based closeness. The program is psychoeducational and teaches skills such as teamwork, conflict management and communication. It has eight sessions throughout the perinatal period and is run in groups. The program has been tested in multiple studies, revealing promising effects for mothers, fathers and children. Outcomes include: decreased depression and anxiety for mothers, higher co-parenting support for both parents, higher parenting-related closeness for fathers, more adaptive duration of orienting and soothability for infants, better relationship quality for parents and less child internalizing problems up to 7 years later (Feinberg and Kan, 2008; Feinberg et al., 2014; Jones et al., 2018a).

The initial study (Feinberg and Kan, 2008) found significant effects on coparental support (mother report, *d* = 0.35; father report, *d* = 0.54), father reported parenting-based closeness (*d* = 0.44), maternal depression (*d* = 0.56), maternal anxiety (*d* = 0.38), father reported parent-child dysfunctional interaction (*d* = 0.70), and infant duration of orienting (*d* = 0.34). Effects were not found for paternal mental health or coparental undermining. The follow-up study (Feinberg et al., 2014) found that 6 years later there were significant effects on teacher reported internalizing problems (*d* = 0.55) and boys’ externalizing problems (*d* = 0.75), but no effects for parent reported outcomes. Another larger study of the program (Jones et al., 2018a) found significant effects at 2-year follow-up on triadic relationship quality (*d* = 0.39), negative coparenting (*d* = 0.38), negative parenting (*d* = 0.41), child internalizing problems (*d* = 0.19) and child night wakings (*d* = 0.30).

*Baby Triple P* (Tsivos et al., 2015) is a targeted program for mothers with postnatal depression that uses parenting education. The content includes: infant care strategies, parent coping skills, catching unpleasant thoughts and emotions, breathing, positive self-talk, partner and social support, parenting routines, communication, transition to parenthood and goal setting. There are eight sessions and homework tasks to complete between sessions.

A study including women who had been diagnosed with postnatal depression did not find any significant differences in parent or child outcomes as a result of the intervention (Tsivos et al., 2015). However, the program displayed high acceptability levels with participants. Another study of the program (Popp et al., 2019) took a universal approach and included both mothers and fathers in the program. The study found that babies in the intervention group were more frequently awake and content (*d* = 0.88) and had less inconsolable crying (*d* = 0.85) than those in the control group. Effects were not found for infant behavioral problems or parental outcomes.

*What Were We Thinking* is a universal psychoeducational program for couples and their babies (Fisher et al., 2010). It is delivered in a group setting through one session after birth, with additional take-home materials. The aim of the program is to prevent depressive and anxiety disorders. The program addresses caring for the baby, managing infant crying, and having a healthy mother and father relationship.

A study found effects for the intervention on mental health, but only among mothers with no psychiatric history (Fisher et al., 2010). For these mothers, the intervention was associated with a significantly lower risk of being diagnosed with depression, anxiety or adjustment disorder, the relative risk reduction being 48%. In a later study, the intervention has also displayed effects on self-rated health (Fisher et al., 2016). No effects have been found for unsettled infant behaviors, mother-baby relationship quality or partner relationship satisfaction (Fisher et al., 2016).

## Discussion

In this review, we found a total of 27 interventions for infants and young children aged 0–3 and their parents, with these spanning a wide range of approaches and target outcomes. Programs were universal or targeted, with some being delivered as both across different studies (Tsivos et al., 2015; Popp et al., 2019). Interventions were focused on outcomes such as maternal mental health, parenting skills, coparenting, parent-infant attachment, reflective functioning, child wellbeing and the couple relationship. Some programs focused on multiple outcomes. The programs also differed in the approaches they used, with these including cognitive behavioral therapy, mentalization based therapy, psychoeducation, practical skills training, and mindfulness.

McLuckie et al. (2019) conducted a scoping review of mental health programs for 0–5 year old children, exploring the overall view of existing interventions, including a broad summary of therapeutic mechanisms, outcome measures, geographical location, research designs, levels of intervention and target populations. The researchers found that the largest proportion of programs were selective, while there were far lower numbers of universal programs. The most common approaches utilized were parenting education groups, interventions targeting the parent-child dyadic relationship and home visits (McLuckie et al., 2019).

We took a similar approach in this review, although we focused on a more specific age group and explored the content of individual programs. In our review, we focused on the 0–3 age group, as this is a critical time of development in which early influences can greatly shape a child’s future trajectory (Zero to Three, 2012; Lyons-Ruth et al., 2017). Following on from the scoping review (McLuckie et al., 2019), we aimed to examine individual programs that currently exist, in order to determine the approaches that have been effective at targeting mental health in this age group.

### Universal and Targeted Programs

Universal and targeted programs have both displayed evidence of efficacy for child and parent outcomes. Targeted programs appear to be particularly effective for maternal mental health outcomes. Programs targeted for mothers at higher risk – such as PREPP, the ROSE Program, Mother and Babies, Mindful Motherhood, Mom Power and the antenatal group program – have demonstrated multiple impacts on mental health outcomes such as symptoms of depression, anxiety and PTSD, risk of a clinical diagnosis, stress, negative affect and mood regulation (Zlotnick et al., 2001; Vieten and Astin, 2008; Thomas et al., 2014; Muzik et al., 2015; Werner et al., 2016; Tandon et al., 2018). Although targeted programs have also demonstrated some effects on other outcomes, the majority of effects appear to be related to maternal mental health.

Universal programs are able to impact maternal mental health outcomes too, yet also appear to have wider effects on other outcomes. A number of universal programs – Family Foundations, Toward Parenthood, What Were We Thinking, and Couple CARE – appear able to influence outcomes across a number of domains, including parental mental health, infant mental health, the parenting relationship, and the parent-child relationship (Feinberg and Kan, 2008; Fisher et al., 2010; Halford et al., 2010; Milgrom et al., 2011). Universal programs have the added advantage that they are able to reach a larger proportion of the population.

Family Foundations, in particular, is a universal program that has demonstrated effectiveness on outcomes for both parents and children. It is one of the programs with the longest term effects, with reduced child internalizing symptoms evident up to 6 years after the program was delivered (Feinberg et al., 2014). The studies on this program have been of high quality, using randomized controlled trials with sample sizes of a few hundred participants.

### The Parent-Child Relationship

Considering the importance of the parent-child relationship that has been highlighted in the literature, we explored the programs that focused enhancing secure attachment and parental reflective functioning. In line with the scoping review of McLuckie et al. (2019), we found that a large proportion of current intervention programs focus on the parent-child relationship.

The AMPLE program was effective at increasing emotional availability for young mothers (Nicolson et al., 2013). Studies on the PALS program have found results for maternal and child behavioral outcomes, especially for high risk women (Landry et al., 2008). The Lighthouse Parenting Program was able to improve parental self-efficacy (Byrne et al., 2018). Minding the Baby has been effective at improving maternal reflective functioning and child attachment (Sadler et al., 2013; Slade et al., 2020). The HUGS program was able to improve bonding and maternal communication with infants (Holt et al., 2021) and reduce parenting stress (Milgrom et al., 2006).

Mom Power appears to be a promising program which has demonstrated efficacy for maternal mental health symptoms and other outcomes across four studies, two of which were randomized controlled trials (Rosenblum et al., 2018). A large number of studies have been conducted on the Circle of Security intervention. Many of these were intervention only studies, which makes it difficult to determine if results were due to the intervention (Rose et al., 2018). However, randomized controlled trials of the program have also indicated that it is effective in improving parenting and child attachment (Cassidy et al., 2011; Mothander et al., 2018).

The PEEP program, Families First, and the universal attachment skills program also focused on building the parent-child relationship. However, the efficacy of these programs is hard to determine. Families First does not yet have any published data, and the data for the PEEP program and the universal attachment skills program was not analyzed in a way that enabled statistical conclusions to be drawn.

Considering that attachment and reflective functioning based programs have demonstrated some effects for parents and their children, it appears that targeting aspects of the parent-child relationship is an important feature to include in interventions. It is worth noting that the research on Mom Power, AMPLE, PALS, Minding the Baby, HUGS and the Lighthouse Parenting Program has been on targeted samples, while Circle of Security has been trialed as both a universal and targeted program. The majority of these studies only involved mothers, rather than both parents. In future studies, it would be beneficial to explore the effects of attachment and reflective functioning programs on universal samples, and on targeted samples which include both parents, as this is a gap in the literature.

### The Parent Couple Relationship

As well as the parent-child relationship, the relationship between parents has also been identified in the literature as an important factor for child mental wellbeing. Programs that specifically targeted the parenting relationship were Couple CARE for Parents, Family Foundations, and What Were We Thinking. Couple CARE has demonstrated a number of effects, particularly on couple communication (Halford et al., 2010). However, some of the program’s effects were only found for mothers. Family Foundations has been effective in improving parent mental health, the couple relationship and child outcomes (Feinberg and Kan, 2008; Feinberg et al., 2014). What Were We Thinking has demonstrated mixed results; it is possibly effective at improving mental health outcomes, but does not seem to impact the parenting relationship or infant outcomes (Fisher et al., 2010, 2016). All three of these programs have been tested in universal studies, while Couple CARE has also been run as a targeted intervention.

Baby Triple P is a program for mothers that has recently been studied when run in a couples format (Popp et al., 2019). Results were found for infant outcomes but not for parenting outcomes. Of the programs targeting the couple relationship and coparenting, Family Foundations and Couple CARE for Parents appear to be the most promising (Feinberg and Kan, 2008; Petch et al., 2012). Couple Care for Parents appears effective at improving outcomes related to the relationship between partners, while Family Foundations has demonstrated effects on the parenting relationship and mental health for parents and children. Both of these programs are psychoeducational and involve running through six to eight sessions as a couple with a trained facilitator. They have the advantage of being relatively brief and cost effective to implement.

Four of the programs – Toward Parenthood, the antenatal group program, Families First, and Minding the Baby – encouraged partners to attend some of the sessions, but did not have the couple relationship as a specific focus of the intervention. This appears to be a common finding in the literature. A review of partner inclusive interventions found that nine out of thirteen studies addressing the parenting relationship appeared effective in reducing depression and anxiety (Pilkington et al., 2015). However, the majority of these did not actually test the effectiveness for partners, and some did not even involve partners, despite including content on the parenting relationship (Pilkington et al., 2015). Given the consistent evidence of the importance of partner support, this needs to be taken into account when developing future interventions (Pilkington et al., 2015).

### The Role of Fathers

While the programs previously mentioned included both parents, there is a shortage of programs that specifically focus on helping fathers to transition to the parenting role. Programs tend to focus on the mental health of mothers, and do not include fathers and partners (Pilkington et al., 2015; O’Brien et al., 2017). Given the link between the mental health of mothers and partners, and the influence of the father on the infant and young child’s development, this is an important gap to address.

Boot Camp for New Dads was the only program reviewed that was designed specifically for fathers, to help them bond with their baby (Capuozzo et al., 2010). It appears that the program was effective, however, the results were not analyzed statistically, so this is difficult to determine with certainty. More research needs to be conducted, especially to investigate the effects of this program on mental health for fathers. Focusing interventions on the mother–child attachment relationship alone is not sufficient, and further research should be conducted on the impact of these interventions on father-child relationships, as well as fathers’ mental health.

### Programs Aimed at Children

While the majority of programs involved the parents as participants, the Responsive Early Childhood Curriculum, the Relationships for Growth and Learning Program and Early Head Start focused mainly on children (Love et al., 2005; Landry et al., 2014; Bekar et al., 2017). These programs were targeted for toddlers at risk, due to individual or environmental risk factors. Relationships for Growth and Learning used principles of play psychotherapy and was successful in that it enabled at-risk toddlers to catch-up to their peers in behavioral functioning (Bekar et al., 2017). Early Head Start has been studied extensively with 3,000 families, with the research finding that it has positive impacts on children’s cognitive, behavioral, social and emotional functioning (Love et al., 2005). The Early Head Start programs were most effective when delivered in both home and community settings. The RECC focused on building responsive interactions with toddlers, and found results for social and emotional competence (Landry et al., 2014). It has been studied with a large sample of toddlers.

### Qualitative Experiences

Most of the research we found was quantitative in nature. However, studies would also benefit from collecting qualitative data. Qualitative results can illuminate effects of the program that are missed by quantitative data. For example, the Lighthouse Parenting Program interviewed mothers who participated in the program. Although the program did not achieve all of the expected outcomes, the discourse from mothers emphasize its value to participants. It would be beneficial for future studies to explore qualitative experiences of parents in the program.

## Conclusion

Pregnancy and infancy are important developmental periods where the mental wellbeing of both parents and infants needs to be considered in order to prevent the emergence of mental health difficulties during infancy and early childhood. Research has demonstrated that it is possible for infants and children aged from zero to three to develop mental disorders, which must be addressed and treated (Warner and Pottick, 2006). Influences in the early developmental years of a child’s life have the capacity to affect them into adulthood. For this reason, there is scope for early interventions during this period to set children up for a positive future life trajectory. Furthermore, rates of parental depression and anxiety during the perinatal period emphasize the need for interventions to address mental health during this time (Woody et al., 2017; Philpott and Corcoran, 2018).

A number of interventions for parents and children have already been researched. The literature suggests that programs that target the parent’s mental health and wellbeing, as well as the parent-child and couple relationship have the potential to promote good parent and child mental health. Our findings suggest that mothers, partners and children should all be included for an intervention to be most effective. Parent interventions ideally focus on improving the parents’ mental health as well as building protective factors that will benefit their children. As demonstrated in a range of studies already described, interventions for parents and children have the capacity to build protective factors including secure attachment, parental mentalizing capacity and a positive coparenting relationship.

The interventions described above can act as a guide for future intervention efforts during pregnancy and infancy. Programs that were based on cognitive-behavioral principles showed evidence of efficacy (Milgrom et al., 2011; Petch et al., 2012; Tandon et al., 2018). Practical skills training about how to care for the baby was another feature of effective programs (Fisher et al., 2010; Werner et al., 2016). A number of programs considered attachment or parent-infant bonding (Capuozzo et al., 2010; Maskell-Graham, 2014; Kalland et al., 2016; Werner et al., 2016; Tandon et al., 2018). *Families First* and the *PEEP Reflective Parenting Program* did this through building parental reflective functioning capacity (Maskell-Graham, 2014). The coparenting relationship between parents was another effective component (Feinberg and Kan, 2008; Fisher et al., 2010; Petch et al., 2012), as was the importance of building wider support networks (Zlotnick et al., 2001; Milgrom et al., 2011; Maskell-Graham, 2014; Kalland et al., 2016). Research also suggests that interventions involving children aged zero to three are effective for promoting healthy development. This may be particularly important for infants who come from a high risk background. The interventions for infants described in this review were targeted at high risk infants and displayed evidence of efficacy.

The research evidence suggests that the theoretical backgrounds of interpersonal therapy, cognitive-behavioral therapy, attachment theory and mentalization are ideal starting points for program development. A review of available interventions highlights the need to pull together all of the successful elements of the available interventions and attempt to combine them in an effort to establish an intervention that aims to improve parental mental health and prevent difficulties, support the couple relationship and develop secure parent child relationships which in turn will improve infant mental health. Furthermore, many of the studies, even when aimed to improve child outcomes, did not measure or report these outcomes to evaluate if the intervention indeed had the desired outcome. Future studies should include control groups for the comparison of outcomes. They should also measure the mental health of both parents, attachment style and parental reflective functioning, childhood outcomes and the quality of the couple relationship.

In summary, there is great potential to intervene during pregnancy and infancy to improve the mental health of both infants and their parents. Early life mental health difficulties can continue to impact the individual well beyond this time, and thus it is important to develop interventions that can prevent these difficulties. As outlined in this review, several programs have already been developed with this goal in mind. Knowledge of the effective components of these interventions can be used to inform the development of future prevention efforts, with the aim of optimizing mental health outcomes for infants and their families. The mental health team of the ORIGINS Project, in partnership with Telethon Kids Institute, is currently developing a program that addresses these outcomes, with the aim of enhancing prevention efforts during infancy and early childhood. The program will be piloted at a large health campus located in Perth, to assess the efficacy for children and their parents.

## Author Contributions

RR conceptualized the scope of the review and was the lead researcher in the project. EI researched the topic and wrote article content. SP is the director of the ORIGINS Project, to which this review belongs; she contributed content to the review. MD and MM researched the topic, wrote article content, and prepared the manuscript. AB worked on editing the manuscript. All authors contributed to the article and approved the submitted version.

## Conflict of Interest

The authors declare that the research was conducted in the absence of any commercial or financial relationships that could be construed as a potential conflict of interest.

## Publisher’s Note

All claims expressed in this article are solely those of the authors and do not necessarily represent those of their affiliated organizations, or those of the publisher, the editors and the reviewers. Any product that may be evaluated in this article, or claim that may be made by its manufacturer, is not guaranteed or endorsed by the publisher.
